# Positive and negative hysteresis effects for the perception of geometric and emotional ambiguities

**DOI:** 10.1371/journal.pone.0202398

**Published:** 2018-09-26

**Authors:** Emanuela Liaci, Andreas Fischer, Harald Atmanspacher, Markus Heinrichs, Ludger Tebartz van Elst, Jürgen Kornmeier

**Affiliations:** 1 Institute for Frontier Areas of Psychology and Mental Health, Freiburg, Germany; 2 Section for Experimental Neuropsychiatry, Department for Psychiatry & Psychotherapy, University of Freiburg, Freiburg, Germany; 3 Faculty of Medicine, University of Freiburg, Freiburg, Germany; 4 Department of Psychology, University of Freiburg, Freiburg, Germany; 5 Collegium Helveticum, Zürich, Switzerland; Technical University of Madrid, SPAIN

## Abstract

**Aim:**

The present study utilizes perceptual hysteresis effects to compare the ambiguity of Mona Lisa’s emotional face expression (high-level ambiguity) and of geometric cube stimuli (low-level ambiguity).

**Methods:**

In two experiments we presented series of nine Mona Lisa variants and nine cube variants. Stimulus ambiguity was manipulated by changing Mona Lisa’s mouth curvature (Exp. 1) and the cubes’ back-layer luminance (Exp. 2). Each experiment consisted of three conditions, two with opposite stimulus presentation sequences with increasing and decreasing degrees of ambiguity, respectively, and a third condition with a random presentation sequence. Participants indicated happy or sad face percepts (Exp. 1) and alternative 3D cube percepts (Exp. 2) by key presses. We studied the influences of a priori perceptual biases (long-term memory) and presentation order (short-term memory) on perception.

**Results:**

Perception followed sigmoidal functions of the stimulus ambiguity morphing parameters. The morphing parameter for the functions’ inflection points depended strongly on stimulus presentation order with similar effect sizes but different signs for the two stimulus types (positive hysteresis / priming for the cubes; negative hysteresis / adaptation for Mona Lisa). In the random conditions, the inflection points were located in the middle between those from the two directional conditions for the Mona Lisa stimuli. For the cube stimuli, they were superimposed on one sigmoidal function for the ordered condition.

**Discussion:**

The hysteresis effects reflect the influence of short-term memory during the perceptual disambiguation of ambiguous sensory information. The effects for the two stimulus types are of similar size, explaining up to 34% of the perceptual variance introduced by the paradigm. We explain the *qualitative* difference between positive and negative hysteresis with adaptation for Mona Lisa and with priming for the cubes. In addition, the hysteresis paradigm allows a *quantitative* determination of the impact of adaptation and priming during the resolution of perceptual ambiguities. The asymmetric shifts of inflection points in the case of the cube stimuli is likely due to an a priori perceptual bias, reflecting an influence of long-term memory. Whether corresponding influences also exist for the Mona Lisa variants is so far unclear.

## Introduction

### Perceptual ambiguities

Our senses have only limited access to the environmental information and the available sensory evidence can be to varying degrees ambiguous (e.g., [1]). Our perceptual system needs to disambiguate and interpret the available sensory information in order to construct stable and reliable percepts. Ambiguity occurs in different sensory modalities (e.g., [2–4]) and at different processing levels, reaching from low-level figure-ground segregation (e.g., [5]) or depth processing (e.g., [6]), over motion integration (e.g., [7,8]) to the emotional content of faces (e.g., [9]) or semantic and linguistic ambiguities [10]. Ambiguities in perception, action, and communication can be the source of misunderstandings and conflicts.

Perceptual ambiguity implies that one and the same sensory information allows for two or more interpretations. Classical ambiguous figures, like Rubin’s vase/face figure [5], Boring’s old/young woman [11], the Necker cube (ambiguity in depth perception [6]) and binocular rivalry stimuli (e.g., [12]) have been used to study ambiguity at lower processing levels (e.g., [13–17]). They have been in the focus of scientists from disciplines like philosophy, cognitive and neuroscience and even physics [15–19] for decades.

**Fig 1:**
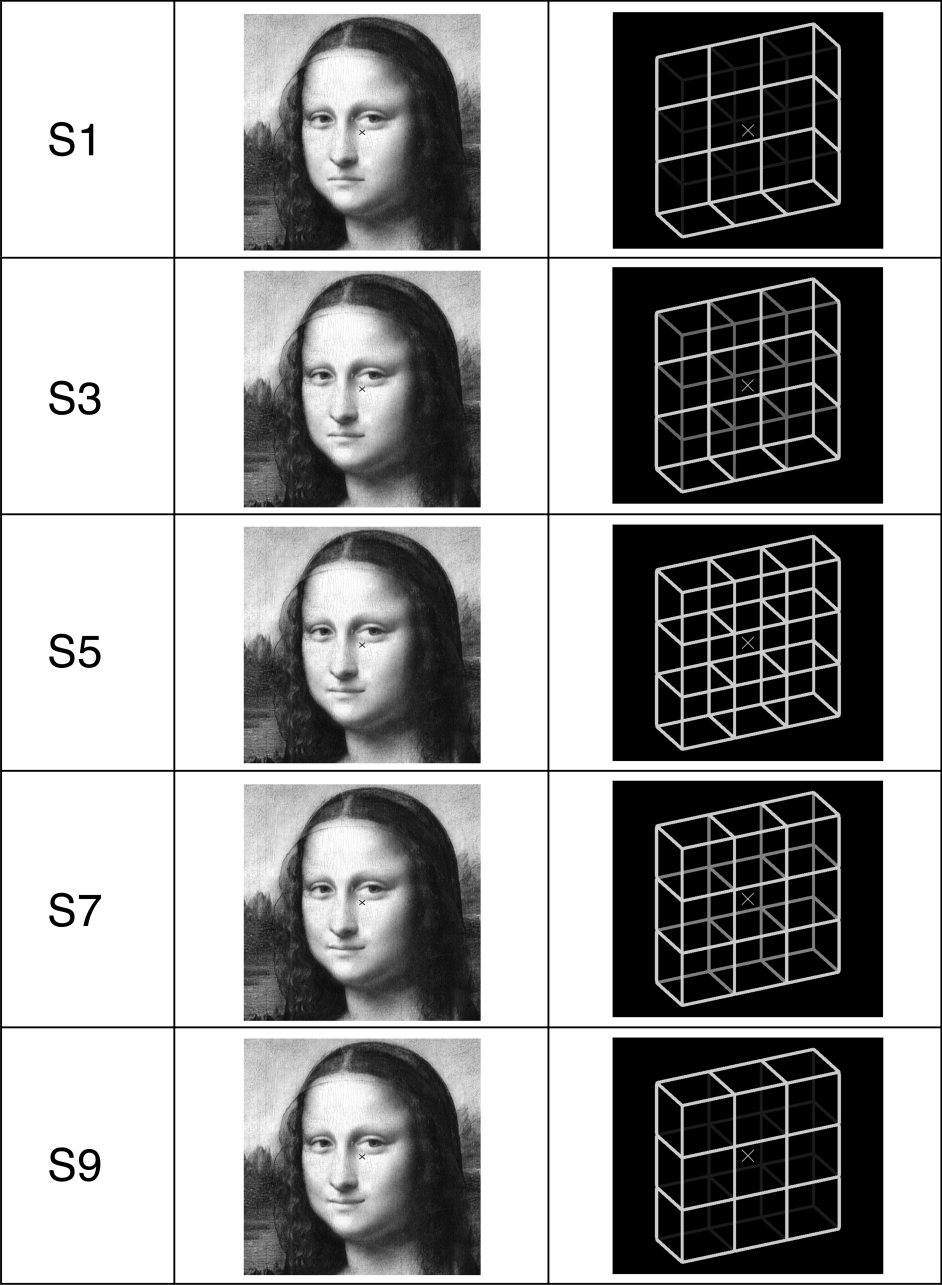
Five of the Mona Lisa and Necker lattice variants (left column, S1, S3, S5, S7 and S9) used in Experiments 1 and 2.

The Necker cube, as one of the most studied ambiguous figures, is a two-dimensional projection of a 3D wire cube. Its physical information entering the retinae allows in principle for infinitely many interpretations (see Fig 2C in [20]), yet our perception is mainly binary, flipping back and forth between two interpretations with presupposed 90° angles for all corners, i.e. a front-side-down- and a front-side-up-perspective (see the ambiguous Necker lattice, a combination of nine Necker cubes in the right column in Fig 1, S5, and disambiguated variants S1 and S9 thereof). Moreover, there is a slight preference in favor of the **f**ront-side-**d**own (**FD**: Fig 1, S9) over the **f**ront-side-**u**p interpretation (**FU**; Fig 1, S1; e.g., [20–23]). This perceptual bias may be explained by the fact that in our everyday life we look more often down than up at objects, and that they are mostly illuminated from above (e.g. by the sunlight or light bulb) rather than from below. It thus indicates an important role of a perceptual statistics, stored in long-term perceptual memory (LTM), while resolving the Necker cube ambiguity.

Further evidence indicates that the immediate perceptual history, stored in short-term memory (STM), can also influence the disambiguation process. If one first observes a disambiguated cube variant, perception of the subsequently presented cube tends to be biased towards a percept in line with the previous one (priming) or opposite to it (adaptation). Several studies have shown that adaptation and priming play significant roles during perception of ambiguous stimuli (e.g., [24–31]).

Ambiguities can also occur at higher processing levels, e.g. when we look at the face of a person in front of us and, in particular, when we try to interpret her/his emotional state from her/his facial expression. Associating certain features of the face pattern with certain emotional states of an observed person requires us to apply social cognition and theory-of-mind concepts because the reference system for emotional states is endogenous in nature and is based on our own memorized first-person experiences of emotional states (e.g., [32–34]).

One of the most prominent examples of a stimulus with ambiguity in emotional face expression is Leonardo da Vinci’s Mona Lisa (around 1503–1507). Mona Lisa’s emotional expression has been acknowledged worldwide for being enigmatic, offering a persistent ambiguity of a happy face at one moment, fading towards a melancholic expression a moment later [35,36]. Similar to the a priori perspective bias in the case of the Necker cube, we recently identified an a priori happiness-bias (LTM effect), as well as an influence of the immediate perceptual history (STM effect) during perceptual disambiguation of the emotional face content of Mona Lisa variants [9]. In addition, several studies demonstrated priming and adaptation in the perception of emotional face contents [28,37–46].

Despite these similarities, there are obvious differences between the two types of ambiguity: With Mona Lisa we do not experience the clear-cut reversals between two perceptual interpretations as with the Necker cube. Instead, the perceived intensity of happiness or sadness can vary gradually within and between observers over time. Further, the Necker cube is a “physically ambiguous reference stimulus”, in the sense that all possible interpretations are physically equally plausible. In contrast, our recent evidence suggests that in the case of Mona Lisa no such clearly defined physically ambiguous reference stimulus exists [9].

From a broader perspective, perception of classical ambiguous figures like the Necker cube [6] or Borings old/young woman [11] has been considered as low-level model for cognitive decision processes in higher-level planning and/or executive cognitive modules (e.g., [18,47–49]) and for an interplay between bottom-up sensory infor-mation and top-down concepts from memory and/or attentional functions (e.g., [50,51]).

In line with these views, recent evidence from our studies indicates that some steps in the neural processing of sensory ambiguity occur at a very abstract level and are generalized across visual categories [52,53]. However, it is still unclear whether and how lower-level ambiguities of classical and artificial ambiguous figures are related to ambiguities at the higher level of emotional face contents.

In the present study, we systematically investigated parallels and differences across these levels of ambiguity. We used a perceptual hysteresis paradigm in order to compare the role of short- and long-term memory during perceptual disambiguation of the two stimulus categories.

### The percptual hysteresis paradigm

Hysteresis effects were first discovered for the behavior of magnetic materials in the late 19^th^ century. Broadly speaking, hysteresis characterizes the dependence of a system’s state on its history. Similarly, any current perceptual state depends to some degree on previous, memorized perceptual experiences. Perceptual hysteresis effects have been described for flicker-fusion thresholds [54], in the representation of affordances [55–58], and for ambiguities in perception [23,59–64].

Importantly, experimental hysteresis paradigms in perception (see Methods) provide quantitative estimates of memory contributions from different time scales to the perceptual disambiguation of ambiguous stimuli [23]. This paradigm may thus be a suitable tool to investigate commonalities and differences of ambiguity resolution across processing levels.

**Fig 2:**
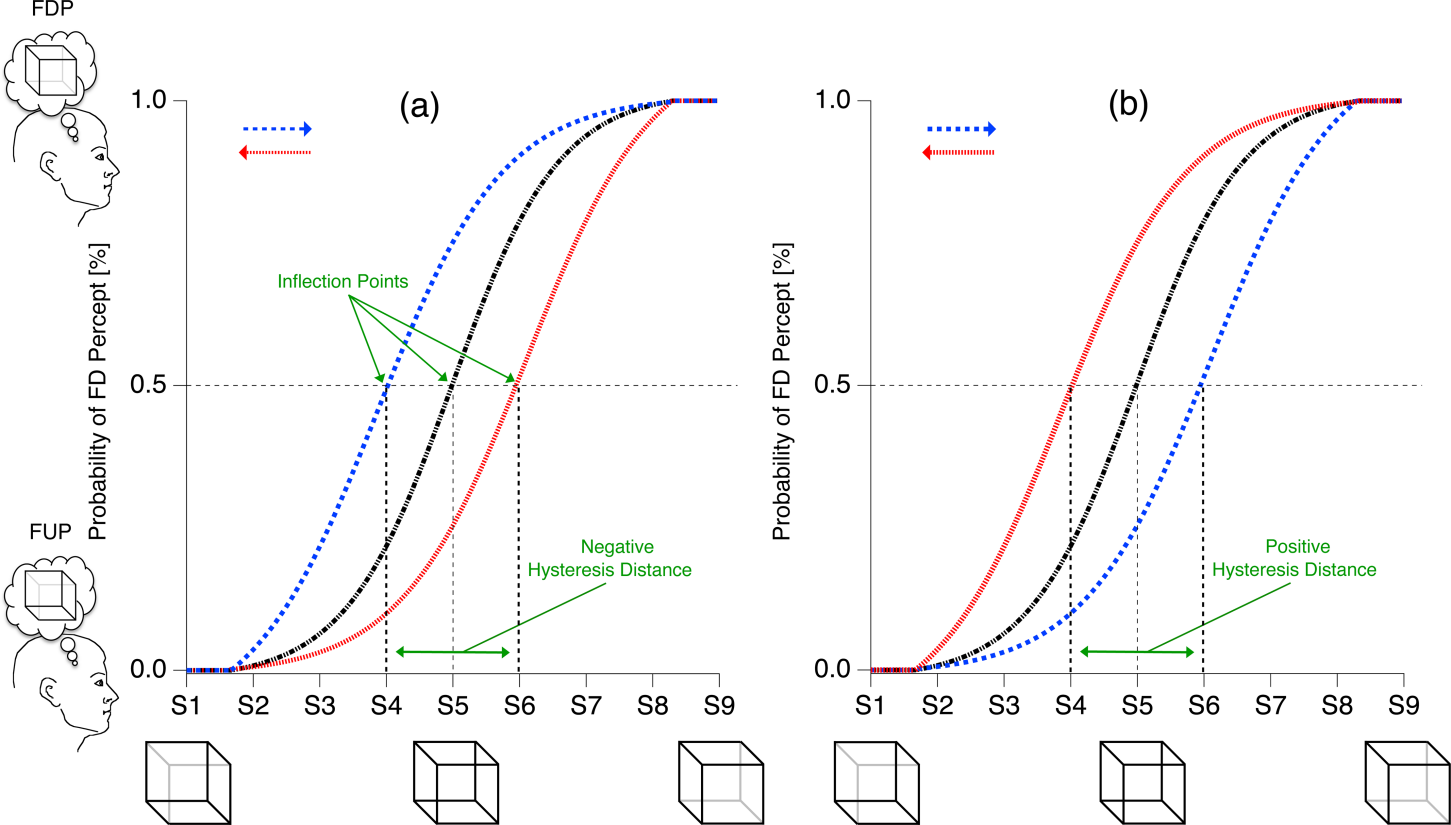
Perceptual hysteresis effect. Perception (ordinate) changes as a sigmoidal function of changing luminance S of the cube’s back-layer as stimulus morphing parameter. The hysteresis paradigm consists of two conditions (red and blue traces) with sequential stimulus presentation, with a stepwise increase (from S1 to S5) and thereafter a decrease (S5 to S9) of stimulus ambiguity. The resulting sigmoid functions change as a function of stimulus presentation order (blue trace: starting with S1; red trace: starting with S9 as indicated by the blue and red arrows). The horizontal distance between the inflection points of the two (blue and red) curves is the hysteresis distance and reflects the influence of immediately past perceptual history, stored in STM. Presenting the stimuli S1 to S9 in random sequence and averaging across repetitions eliminates the influence of STM. The resulting inflection point should thus be located at Sa related to the physically most ambiguous reference cube (Necker cube, Sa = S5). (a) *Short-term memory with adaptation impact*. The ordered stimulus presentation shifts the sigmoid function toward the direction opposite to stimulus presentation order, with respect to the random condition (black trace). In this case perception already alternates even though the observed stimulus still contains cues favoring the initial percept (S4 for the blue trace and S6 for the red trace). According to a predefined calculation rule (inflection point value from the blue minus inflection point value from the red trace), the hysteresis distance has a negative sign; therefore the effect is called negative hysteresis. (b) *Short-term memory with priming impact*. The ordered stimulus presentation shifts the sigmoid function toward the direction of the stimulus presentation order, with respect to the random condition (black trace). In this case the percept only alternates when the observed stimulus already contains cues favoring the alternative percept (S6 for the blue trace and S4 for the red trace). The hysteresis distance between the locations of the inflection points for blue minus red traces is positive; therefore the effect is called *positive hysteresis*.

In the perceptual hysteresis paradigm used in the present paper, cube stimuli with different luminance values of the apparent back-layer are sequentially presented. The sequence starts with cube stimuli containing cues in favor of a **f**ront-side **u**p perspective (FUS, variant S1 in Fig 2) with maximally reduced luminance S (stimulus morphing parameter) of the apparent back-layer. Then S is linearly increased up to S_**a**_ (Eqs 1 and 2), related to the physically most **a**mbiguous cube (the Necker cube) with isoluminant edges (Fig 2 variant S5 in Fig 2), followed by back-layer luminances increasingly favoring the alternative **f**ront-side **d**own perspective (FDS, variant S9 in Fig 2). This will lead to a hysteresis effect: Perception will alternate from FUP (**f**ront-side **u**p **p**ercept) to FDP (**f**ront-side **d**own **p**ercept) at a certain back-layer luminance value S_FUP_→_FDP_, which differs from the value S_FDP_→_FUP_ when moving from FDS to FUS (see Fig 2).

In a sample of experimental repetitions, stimulus perception can be expressed as a sigmoidal probability function of the stimulus morphing parameter S, and the function’s inflection point at probability p = 0.5 can be used to estimate the critical morphing parameter values S _FUP_← _FDP_ and S_FUP_→_FDP_ (Fig 2). Different stimulus presentation orders (either starting from the right or from the left in Fig 2) will then lead to horizontal shifts of the corresponding sigmoid functions (blue and red traces in Fig 2) and, thus, to different inflection point values S_FUP_→_FDP_ and S _FUP_← _FDP_.

In the following, we explain how we used the hysteresis paradigm to disentangle short-term and long-term memory contributions to the disambiguation of ambiguous sensory information. For this purpose we only consider the location of the inflection point as a function of the stimulus presentation sequence and disregard the gradient of the sigmoidal function.

In the first step, we replaced the morphing parameters (luminance in case of the cubes) by a scale from 1 to 9, corresponding to the sequence of presented stimuli.

Second, the amount of memory influence on perception can be quantified with the hysteresis paradigm by the amount of horizontal shift of the respective sigmoid functions of perception (see below). We introduce factors F_STM_ (short-term memory factor) and F_LTM_ (long-term memory factor, explained below) indicating how much the stimulus parameter has to be changed in order to compensate for the memory influence. F_STM_ and F_LTM_ are regarded as functionally related to memory contents, whereas the detailed functional relations have yet to be undestood.

Third, we quantified the horizontal shifts of the sigmoid functions by the *hysteresis distance* of their two inflection points (S_FUP_→_FDP_ minus S _FUP_← _FDP_; respectively blue trace minus red trace in Fig 2). This hysteresis distance in ordered stimulus presentations reflects the influence of the immediately preceding percepts, stored in STM, on the current percept. The STM influence (*F*_*STM*_ for the STM influence factor in Eqs 1 and 2) should be directional, i.e. the sign of the hysteresis distance should depend on whether the STM content has priming or adaptation impact (see below for more details). As a consequence, we distinguish between positive and negative hysteresis.

While typical hysteresis effects are positive, recent work has shown that negative hysteresis is far more common than may be expected. Examples of negative hysteresis in laser physics (e.g., [65–69]), and material science (e.g., [70–72]) abound. But negative hysteresis has also received increasing attention in perception, in particular in the field of ecological psychology (see e.g., [56,57,73–78] and also the discussion of multistability later in this paper.

**Fig 3:**
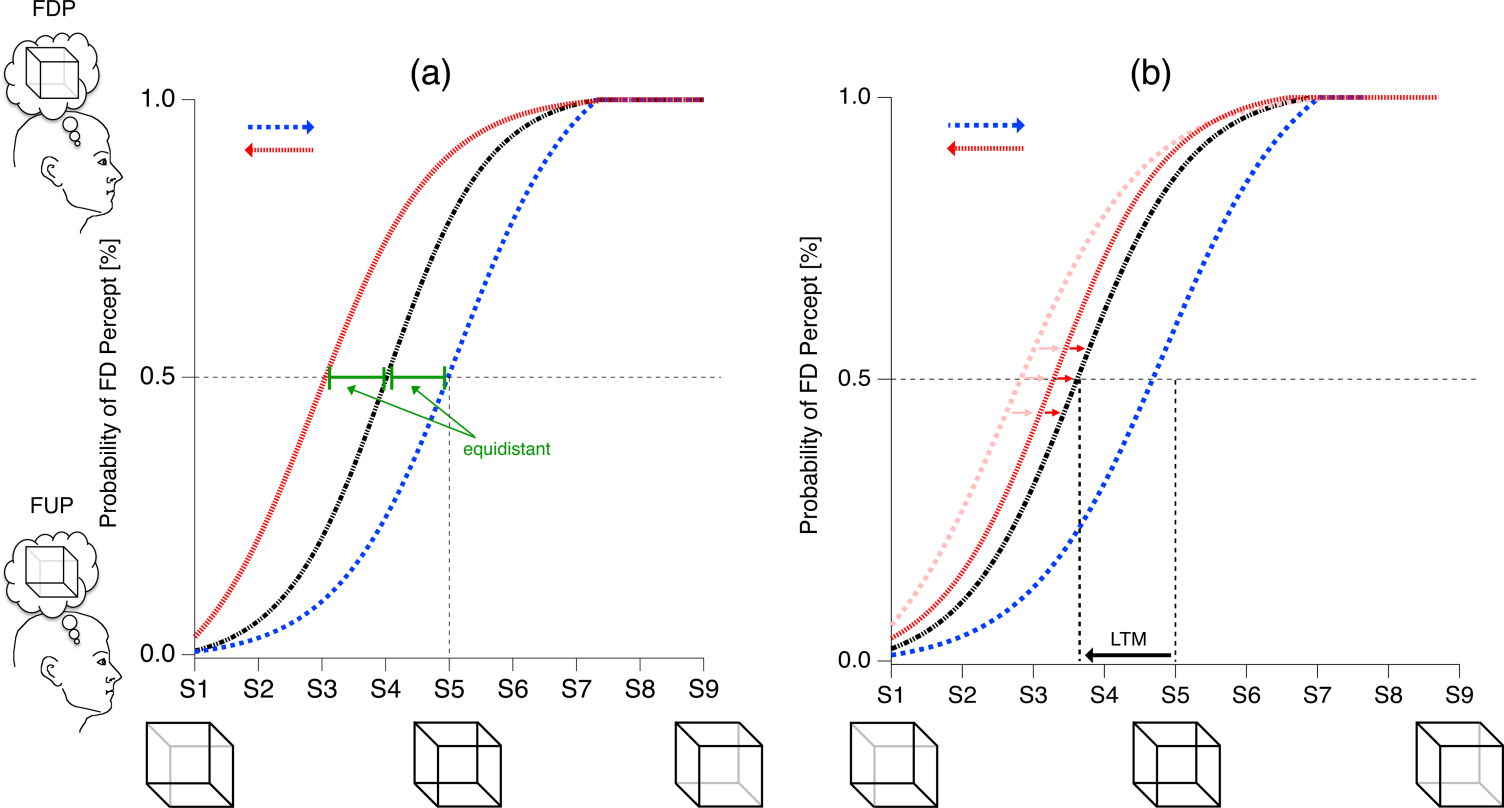
With a randomized stimulus presentation sequence (black trace) the influence of perceptual STM should disappear after averaging across repetitions. Accordingly the inflection point from the random sigmoid function should be located at the physically most ambiguous cube (the Necker cube, Sa = S5), serving as a reference stimulus. However, perception of the Necker cube is known to be biased towards the front-side down perspective (FDP), reflecting the contribution of LTM during perceptual disambiguation. This perceptual bias will be indicated by larger probabilities for cube percepts favoring the FDP interpretation. As a consequence, the inflection point of the related sigmoid function, indicating the perceptually most ambiguous cube variant (between S3 and S4) will already contain 3D cues favoring the non-preferred FUP interpretation. This results in a horizontal shift of the sigmoid function from the random condition (S4 in a and S3 in b, black trace). (a) Assuming an additive impact of FSTM and FLTM during the perceptual process, the sigmoid functions from the two ordered conditions should be shifted by the same amount and in the same direction as the sigmoid function from the random condition. (b) However, there must be a threshold for memory contribution to the perceptual process: If this threshold *S*_*thres*_ is approached by *F*_*LTM*_ (i.e. if the bias is strong enough), the effective influence of STM (*F*_*STM*_ pointing in the same direction as *F*_*LTM*_) on the sigmoid function will attenuate, and *F*_*LTM*_ and *F*_*STM*_ influences become subadditive. The related sigmoid function will then be closer to the one from random presentation order, as indicated by the red shaded traces. In the extreme case of very strong bias, the two sigmoid functions may coincide.

With a randomized stimulus presentation sequence the STM influence should vanish across repetitions because, on average, each cube variant should have each of the other cube variants about equally often as precursor. As a result, the factors *F*_*STM*_ (Eqs 1 and 2), by which the sigmoids are horizontally shifted, should cancel out (Eq 3 and Fig 3A, black traces). In this case the inflection point should be located exactly at the morphing parameter *S*_*a*_ related to the physically most ambiguous reference stimulus with isoluminant edges (*S*_*a*_ = S5 in Fig 3A).

Visual perception can also be influenced by long term memory (LTM), as indicated by the a priori FDP bias in the case of Necker cubes. LTM influence should be independent of the stimulus presentation sequence and should thus uniformly influence both the ordered stimulus presentation sequences (Eqs 1 and 2 and the red and blue traces in Fig 2) and a random stimulus presentation sequence (the black traces in Fig 2). We assume an additive contribution of the factors F_STM_ and F_LTM_ to the location of the sigmoid inflection points in any given hysteresis experiment (Eqs 1 and 2).

$$S_{FUP\leftarrow FDP}=S_{a}+F_{LTM}+F_{STM}$$ Eq 1

$$S_{FUP\to FDP}=S_{a}+F_{LTM}-F_{STM}$$ Eq 2

$$hd=S_{FUP\to FDP}-S_{FUP\leftarrow FDP}=2\times F_{STM}$$ Eq 3

with *S*_*a*_: Stimulus parameter for the physically most ambiguous stimulus; *F*_*LTM*_: LTM factor for horizontal shifts of the sigmoid function; *F*_*STM*_: STM factor for horizontal shifts of the sigmoid function; *hd*: hysteresis distance.

Applying Eqs 1 and 2 reveals that the hysteresis distance “hd” is independent of *F*_*LTM*_ (Eq 3). It reflects twice the influence of STM. Its sign indicates the direction of the STM influence on the position of the inflection points.

A negative hysteresis distance means that the perceptual alternation of cube orientation will occur if the lattice still displays 3D cues, favoring the interpretation before the alternation. This may be interpreted as evidence for adaption (Fig 2A).

A positive hysteresis distance, in contrast, indicates that the alternation of cube orientation occurs only if the related stimulus displays cues favoring the interpretation after alternation. This may be interpreted as evidence for priming (Fig 2B).

Any horizontal shift of the inflection point away from the isoluminant morphing parameter in the random condition (Eq 4 and Fig 3B) indicates that the stimulus needs 3D cues opposite to the preferred interpretation in order to maintain parity between the two interpretations. These 3D cues are then necessary to compensate for the a priori LTM perceptual bias and are expressed by the factor *F*_*LTM*_ in Eqs 1, 2 and 5.

$$S_{Rand}={S_{a}+F}_{LTM}$$ Eq 4

The LTM-induced shift of the inflection point should affect both the random and the two ordered stimulus presentation conditions. Under the assumption of strictly additive contributions of *F*_*LTM*_ and *F*_*STM*_, the sigmoid function from the random stimulus presentation sequence should be located equidistantly from the functions of the two ordered stimulus presentation conditions (see Figs 1 and 2A). The impact of memory on perception, however, must be limited: If the 3D-cues reach a certain threshold strength *S*_*thres*_, they will no longer be “overwritten” by memory contributions. Thus, if the sum of *F*_*STM*_ and *F*_*LTM*_ exceeds *S*_*thres*_, the influences of STM and LTM cannot fully unfold because of a too strong sensory evidence,

$$\left|S_{rand}-S_{FUP\leftarrow FDP}|\neq |S_{rand}-S_{FUP\to FDP}\right|$$ Eq 5

$$for\;|S_{Thres}|<|F_{STM}|+|F_{LTM}|$$

and *F*_*STM*_ and *F*_*LTM*_ will effectively become subadditive. This results in different distances of the sigmoid functions from the two ordered conditions to the sigmoid function from the random condition (Eq 5), such that the symmetry of Fig 3A turns into an asymmetry as in Fig 3B. If the influence of LTM is extreme and drives the inflection point right to *S*_*thres*_, (*F*_*LTM*_ = *S*_*thres*_) the sigmoid function from the random presentation condition will coincide with one of the sigmoid functions from the ordered presentation conditions.

The term “perceptual reversal” is typically used to describe spontaneous changes in perception during the continuous observation of an ambiguous figure. In the present hysteresis paradigm perceptual changes occur across the presentation of stimuli with different degrees of ambiguity with blank-screen gaps between individual stimuli. Given this principle difference between presentation modes we use the term “alternation” instead of “reversal” to describe a change of perception within our paradigm.

The theoretical considerations so far can be summarized in the following way:

1. The contribution of STM during the perceptual disambiguation of ambiguous visual information can be quantified with the hysteresis distance, i.e. the horizontal shifts of the inflection points of the sigmoid functions related to two ordered presentation sequences of ambiguous and disambiguated stimulus variants. A positive hysteresis distance indicates that STM influence is due to priming, a negative hysteresis distance indicates that STM influence is due to adaptation.
2. The contribution of LTM during the perceptual disambiguation of ambiguous visual information can be quantified in two ways, both of which are related to the sigmoid function from randomized presentation order (“the random sigmoid”):
  - by the horizontal shift of the random sigmoid with respect to a physically most ambiguous reference stimulus.
  - by the deviation of the random sigmoid from a position equidistant between the sigmoid functions from the ordered stimulus presentation conditions.

In the present study we applied the hysteresis paradigm to (1) the Necker lattice (Fig 1, S5, right, composed of nine Necker cubes, [79,80]) and disambiguated lattice variants (Fig 1, right, S1, S3, S7 and S9); and to (2) Mona Lisa stimuli (Fig 1, middle column) with different mouth curvatures. The purpose of the study was to compare quantitatively the contributions of STM and LTM to the perceptual disambiguation at these different levels of stimulus ambiguity.

For each stimulus type, we measured both absolute value and sign of the hysteresis distance as an estimate of STM influence. In addition, we measured the location of the sigmoid inflection point from the random stimulus presentation conditions and its position relative to the inflection points from the ordered stimulus presentation conditions, as estimates of LTM influence.

## Methods

### Participants

Thirteen participants (six males, seven females; age range = 20–31, mean age = 26 years) participated in this experiment. Twelve participants were right-handed and one left-handed. All participants were naive as to the specific experimental question and gave their written informed consent. No participants reported any history of neurological disease. Visual acuity was tested via the Freiburg Visual Acuity Test (FrACT, [81]). All participants had a normal or corrected-to-normal vision with right-eye dominance in twelve participants and left-eye dominance in one participant. The study was approved by the ethics committee of the University of Freiburg and performed in accordance with the ethical standards laid down in the Declaration of Helsinki [82].

### Stimuli

**Fig 4:**
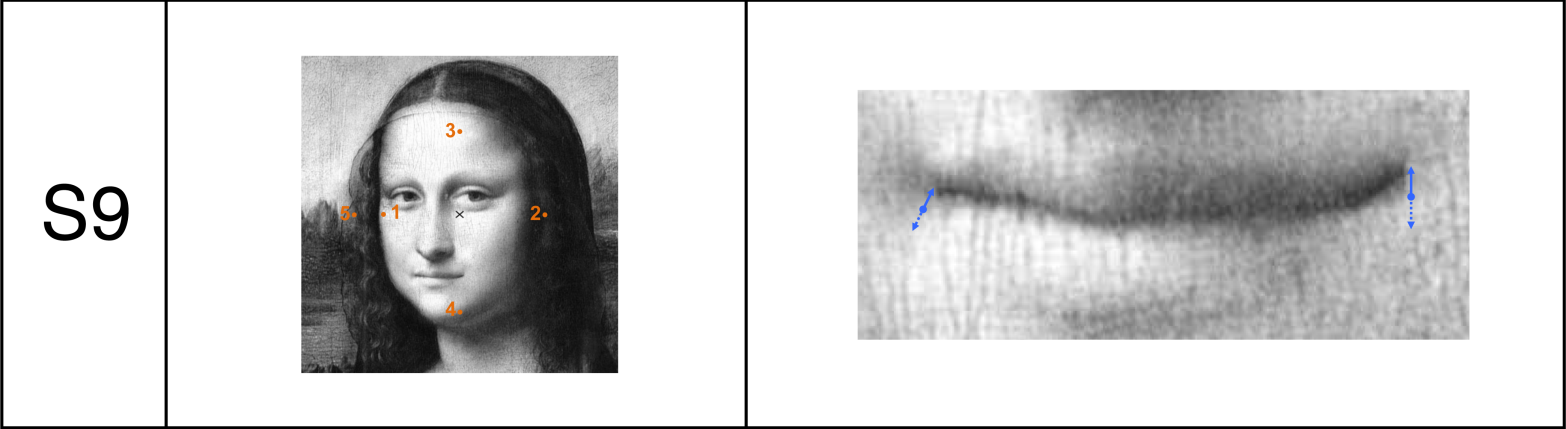
Gray-scale version (S9) of Da Vinci’s original Mona Lisa painting (left) and a magnified excerpt of the mouth region (right). Left: The five orange circles indicate the locations at which luminance was measured. Right: The blue arrows indicate the trajectories of the mouth corner locations of the sadder stimulus variants used in the present experiment. The blue filled circles indicate the left and right mouth corners of the central Mona Lisa S5 variant in the middle of the stimulus range.

In Experiment 1 we used a gray-scale version of Leonardo da Vinci’s Mona Lisa [83] (Fig 1, central column, S9) and created nine variants of it by manipulating the mouth curvature (Fig 4, right column) in order to create a range of emotional face expressions with a stepwise change from sad to happy (Fig 1, central column, S1 –S9).

In Experiment 2 we used an ambiguous Necker lattice [79,80] (composed of nine ambiguous Necker cubes [6]) and eight disambiguated lattice variants covering the range from a most disambiguated **f**ront-side-**d**own **s**timulus (FDS) to a most disambiguated **f**ront-side-**u**p stimulus (FUS), with a stepwise change of the ambiguity level of subsequent stimuli (right column in Fig 1). Lattice disambiguation was realized by stepwise reducing the luminance of the apparent back-layer.

Mona Lisa and lattice stimuli were presented at visual angles of 9.8° × 9.8° and 4.7° × 4.5°. The average of the luminances across five image points (orange points in Fig 4, middle column) for the Mona Lisa stimuli was 27.75 cd/m^3^, whereas for the lattices the luminance values changed in descending order for the back-layer from S6 up to S9 and from S1 up to S4. The luminance values in descending order were: 54.6, 38.2, 19.9, 7.3 and 1.9 cd/m^2^. For all lattice stimuli, the front layer luminance remained at 54.6 cd/m^2^.

### Procedure

In Experiment 1 all nine variants of the Mona Lisa stimulus were presented in three experimental conditions. In the *sad→happy condition* the nine stimulus variants were presented in ordered sequence, starting with the saddest Mona Lisa variant (Fig 1, middle column, stimulus S1, with the least u-shaped mouth curvature) and stepwise increasing happiness up to the happiest Mona Lisa variant (Fig 1, stimulus S9, middle column, with the most u-shaped mouth curvature). In the *happy→sad condition* stimuli were presented in reverse order, starting with stimulus S9 (happiest Mona Lisa) and ending with stimulus S1 (saddest Mona Lisa). In the *random Mona Lisa condition* the nine stimuli were presented in pseudo-randomized order. Participants were instructed to indicate for each presented stimulus in a forced-choice manner either happy or sad face percepts by pressing one of two keys. A third key served to indicate unclear percepts.

In Experiment 2 the nine Necker lattice variants were presented in a similar manner across three experimental conditions. In the *FUS←FDS condition* the nine lattice stimuli were presented in ordered sequence, starting with the FD lattice with the least back-layer luminance (Fig 1, stimulus S9, right column) and stepwise increasing back-layer luminance over the physically most ambiguous lattice variant S5 with isoluminant edges (the Necker lattice, Fig 1, right column), then again reducing luminance of the apparent back-layer up to the FU lattice S1 with the least back-layer luminance (Fig 1, right column). In the *FUS→FDS condition* the nine lattice stimuli were presented in reverse order, starting with stimulus S1 and ending with stimulus S9 (Fig 1, right column). In the *random lattice condition* the nine lattice stimuli were presented in pseudo-randomized order. Participants were instructed to indicate for each presented stimulus in a forced-choice manner either the perception of FU or FD lattice orientations by pressing one of two keys. A third key served to indicate unclear percepts.

Before the start of the two experiments, participants underwent training units, whereby they learned the association between keys and perceptual outcomes. For this purpose, only the most disambiguated variants were presented for each experiment. The training sessions stopped once participants had reached a threshold of at least nine correct responses in a series of ten stimulus presentations for each experiment.

Each experimental condition was repeated ten times and the stimulus order in the *random condition* was newly randomized in each repetition. The conditions of the two experiments were interleaved, i.e. each condition from Experiment 1 was followed by a condition from Experiment 2. The order of conditions within the two Experiments was also randomized. The experimental design was within-participants, i.e. all participants executed all conditions from both experiments.

**Fig 5:**
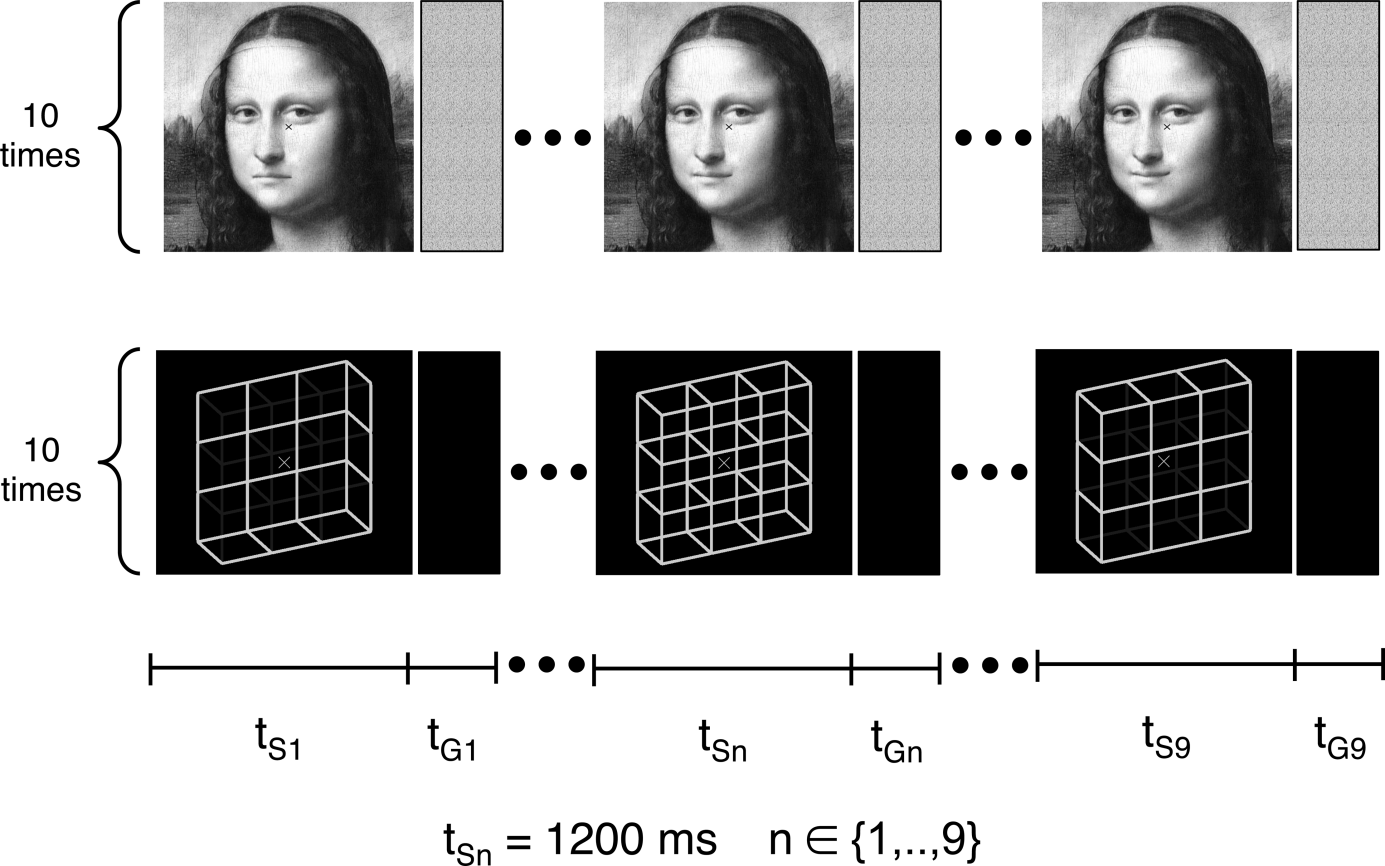
Procedures of Experiments 1 (top) and 2 (bottom). Each stimulus variant was presented for 1200 ms, followed by a screen showing either a gray background similar to the background of the Mona Lisa stimulus (Experiment 1) or a dark gray background identical to the background darkness of the lattice stimuli. S = stimulus, G = gap.

Participants were seated in a chair in a dimly lit room at a distance of 114 cm from the screen. Mona Lisa stimuli were presented for 1200 ms (Fig 5) followed by a 400 ms homogenous gray screen (Experiment 1), comparable to the background gray of the Mona Lisa stimulus. Lattice stimuli were also presented for 1200 ms (Fig 5) followed by a 500 ms homogeneous dark screen. Participants were allowed to respond during the interval from stimulus onset to gap offset. Fig 5 illustrates the experimental protocol for the *sad→happy condition* of Experiment 1 and *FDS→FUS condition* of Experiment 2.

### Analysis

#### Perception

For each participant, condition and stimulus variant from Experiment 1 (Mona Lisa stimuli), we calculated the percentage of happy face percepts (number of happy face responses divided by the total number of responses for the respective stimulus variant). We then sorted the data from all three conditions according to the increase in the mouth curvature’s u-shape (i.e. from S1 = sad to S9 = happy). Further, we fitted psychometric functions to the respective data points for each participant and condition. Then we determined the morphing parameter value of the perceptually most ambiguous stimulus variant (at the 50% happy response level, ordinate), represented by the *inflection point* of the corresponding sigmoidal psychometric function, and its gradient. We used the least-squares fit algorithm (Igor Pro, Wavemetrics) and fixed values for base (= 0) and max (= 1) of the sigmoid fit functions (Eq 6).

$$f(S)=base+\left\{max/(1+\exp\left(\frac{S_{infl}-S}{\left(gradient\right)}\right))\right\},$$ Eq 6

where S is the stimulus morphing parameter and S_infl_ is the S-value at sigmoid inflection point.

Goodness of fit was determined for each participant and experimental condition by calculating R^2^ values. We used inflection points and gradients as dependent variables for repeated -measures ANOVAs, with the factor CONDITION (3 levels, the two directional plus random conditions).

Corresponding analyses were conducted for Experiment 2 with the lattice stimuli.

#### Reaction times

Reaction times were calculated as the time from stimulus onset until the participant’s perceptual response above the predefined threshold of 150 ms. We calculated, separately for each participant, stimulus variant and experimental condition, the mean reaction times across the ten repetitions (for one subject the number of repetitions was accidentally 20 for all three conditions) and entered them as dependent variables in a repeated-measures ANOVA with polynomial contrasts with the factors CONDITION (3 levels) and STIMULUS (9 levels, corresponding to the stimulus variants per experimental condition).

Evidence from the literature indicates a happy face perception bias with faster responses for happy than for sad faces [84–86], a finding recently confirmed with Mona Lisa stimulus variants [9]. Furthermore, after inspecting the reaction time data, we found evidence for a temporal preparation effect: Perception of a stimulus is facilitated and reaction times are faster when the occurrence of that stimulus can be anticipated [87–89]. We post-hoc tested for both, perceptual biases and temporal preparation effects as follows: In the case of Experiment 1 (Mona Lisa) reaction time data from the happiest and from the saddest stimulus variants from the sad←happy and sad→happy conditions were sorted with respect to presentation order (first vs. last). We then calculated a repeated-measures ANOVA with factors STIMULUS (2 levels, most happy and most sad expression) and ORDER (2 levels, stimulus was presented as the first vs. last in the presentation order).

Corresponding analyses of reaction time data were performed for Experiment 2 (lattice stimuli). Perception of the ambiguous Necker cube is known to be biased towards FDP (e.g., [20–23]). Moreover, our lattice reaction time data also demonstrated a temporal preparation effect [87–89]. We thus applied a post-hoc ANOVA for the reaction times in Experiment 2 with factors STIMULUS (2 levels, FDP and FUP, both with maximum difference between front and back layer luminance) and ORDER (2 levels, stimulus was presented as the first vs. last in presentation order), corresponding to the post-hoc ANOVA in Experiment 1.

In each experiment we applied Holm’s variant of the Bonferroni correction for multiple testing [90]. In Holm’s procedure, all calculated p-values are sorted from the smallest to the largest. The first (smallest) p-value is compared with an alpha corrected by the total number n of pairwise comparisons. The second p-value is compared with an alpha corrected by n-1, and so on for the following p-values. All p-values that survived multiple testing corrections are reported.

#### Relations between Mona Lisa and lattice perception

We further tested for differences between reaction times, hysteresis distances and average gradients (across the three different conditions per experiment) from Mona Lisa and lattice stimuli. We also calculated post-hoc correlation coefficients (Pearson and Spearman) between the individual hysteresis distances from the Mona Lisa and the lattice experiment.

## Results

Perception and reaction time data of the individual participants can be found in the supporting information file S1 Data.

### Experiment 1 (Mona Lisa)

#### Perception

The number of`reported unclear percepts was below 5% across all participants.

**Fig 6:**
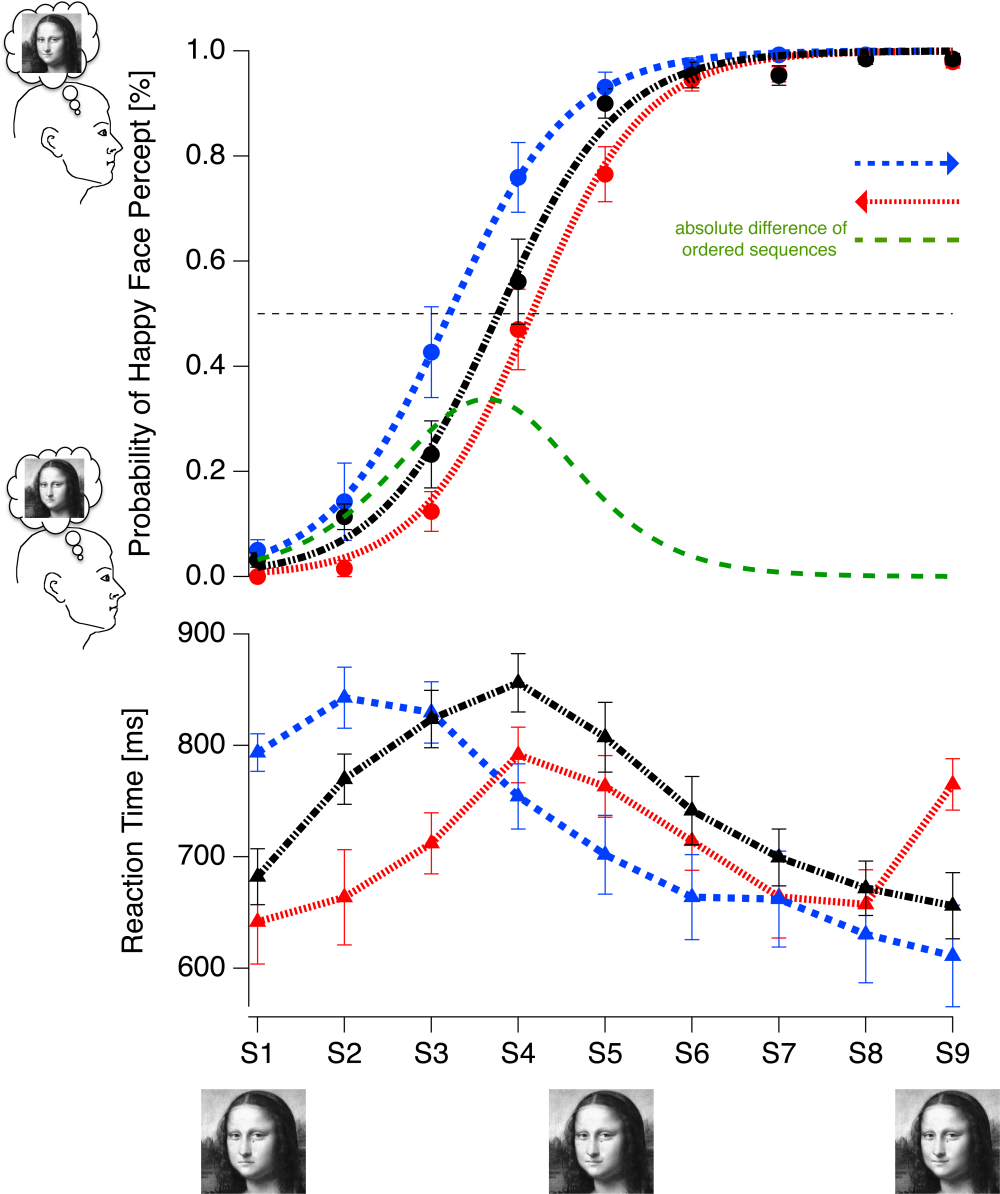
Top graph: Grand mean probability of happy face percepts (filled dots) ± SEM (ordinate) and sigmoidal fits from the *sad→happy* (blue trace), *sad←happy* (red trace) and *random* (black trace) conditions. A clear *negative* hysteresis effect is visible: the sigmoids from the differently-ordered stimulus presentation conditions (indicated in blue and red) are horizontally shifted with respect to one another. The sigmoid from the random condition (black trace) shows a mid position in between the two sigmoids from the ordered conditions. This mid position indicates that memory contribution to the perceptual resolution of ambiguity has not yet reached a threshold. The green difference trace results from a subtraction of the blue trace minus the red trace, indicating the percentage of perceptual variances for individual stimulus variants. Bottom graph: The reaction times (ordinate on the left) related to the perception responses. Reaction times increase with perceptual instability. Furthermore a temporal preparation effect was observed: Less temporal preparation is possible for the first stimulus within an ordered presentation sequence, resulting in longer reaction times compared to the subsequent reaction times.

The perceived emotional expression of Mona Lisa changed as a sigmoidal function of the mouth curvature, as shown in Fig 6 (upper graph). The median range of R^2^s (goodness of fit) across participants and conditions was 0.99 with an interquartile range of 0.02. The repeated-measures ANOVA revealed a main effect of CONDITION (F_(2,24)_ = 21.45, p<0.001) for the variable *inflection point*, indicating that the location of the inflection point changed as a function of stimulus presentation order. No significant effect was found for the variable *gradient*.

**Fig 7:**
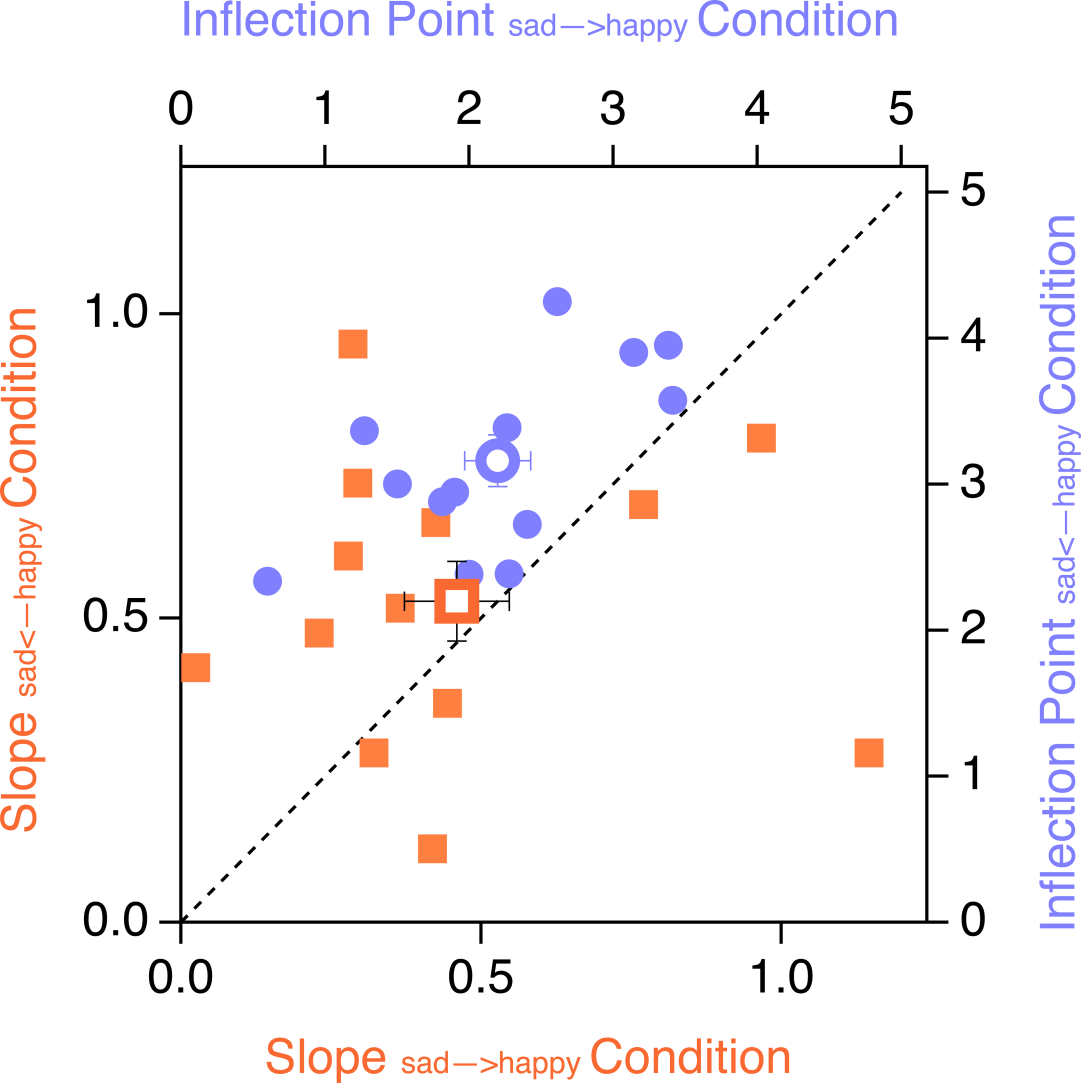
Scatter plot of the sigmoidal fit function parameters of the *sad←happy* and *sad→happy* conditions for each individual participants (filled icons). Orange squares: gradients; cornflower circles: inflection points. Open icons indicate grand means ± SEMs. All cornflower icons are above the bisection line, indicating consistently larger inflection points for the *sad←happy condition* than for the *sad→happy condition*.

The individual inflection points and gradients of the two ordered stimulus presentation sequences are depicted in the scatter plot in Fig 7. All participants had larger inflection points for the *sad←happy* condition compared to the *sad→happy* condition (the respective data points are above the bisection line).

Post-hoc comparisons revealed that the inflection points significantly differed between the three conditions. The inflection points in the *sad→happy condition* were located at a stimulus variant with a less u-shaped mouth curvature (sadder Mona Lisa) than the inflection points in the *sad← happy condition* (happier Mona Lisa) and the inflection points in the *random condition* in between, as can be seen in Fig 7. Bonferroni-Holm corrected p-values from post-hoc t-tests are listed in Table 1.

**Table 1:** Corrected post-hoc t-test results from Experiment 1.

comparison between conditions	p-value concerning the variable inflection point
sad←happy vs sad→happy	0.0018
sad←happy vs random	0.009
sad→happy vs random	0.002

#### Reaction times

In line with the perceptual results, reactions times differed between conditions, as indicated by the main effect for the factor CONDITION (F_(2,11)_ = 5.510, p = 0.011) of the repeated-measures ANOVA (Fig 6, lower graph). Further, reaction times in the sad←happy condition displayed a cubic shape with two local maxima, whereas the sad→happy and the random Mona Lisa conditions displayed a quadratic shape, each with only one maximum. This observation was confirmed by the ANOVA indicating, a quadratic (F_(1,12)_ = 58.2, p < 0.001) and a cubic effect (F_(1,12)_ = 39.5, p < 0.001) for the factor STIMULUS and an interaction between STIMULUS and CONDITION (F_(1,12)_ = 10.63, p < 0.001).

Fig 6 indicates longer reaction times for the first stimuli in the two conditions with ordered stimulus presentations. Further, reaction times seem to be slightly faster for stimulus S9 (happiest Mona Lisa) compared to S1 (sad Mona Lisa). The related post-hoc ANOVA with the factors STIMULUS ORDER and BIAS indicated a main effect of STIMULUS ORDER (F_(1,12)_ = 19.22, p < 0.001) whereas no evidence was found for a happy face BIAS.

### Experiment 2 (Necker lattice)

#### Perception

**Fig 8:**
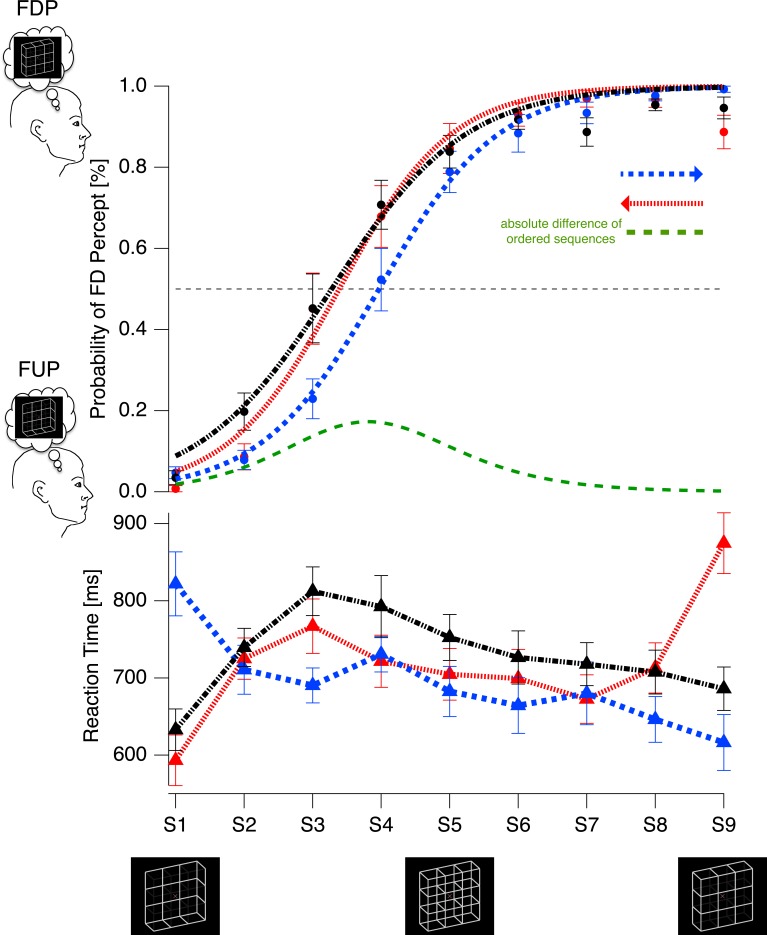
Top graph: Grand mean probability of front-side-down percepts (FDP, filled dots) ± SEM (ordinate) and sigmoidal fits from the *FUS←FDS* (red trace), *FUS→FDS* (blue trace) and *random* (black trace) conditions. A clear *positive* hysteresis effect is visible: The sigmoids from the different ordered stimulus presentation conditions (blue and red traces) are horizontally shifted against each other. There are two remarkable observations: (1) The sigmoid’s inflection point from the random condition is not located at the physically most ambiguous reference stimulus (the Necker lattice, S5) but instead at a stimulus already containing strong FU-cues (close to S3), reflecting the a priori bias from LTM. (2) The sigmoid from the *FUS←FDS condition* (red trace) is superimposed on the sigmoid from the random condition (black trace), indicating that the LTM contribution to the perceptual resolution of ambiguity has already reached a threshold and STM can no longer contribute. The green difference trace results from subtracting the blue trace from the red trace, indicating the percentage of perceptual variance for individual stimulus variants. Bottom graph: Reaction times (ordinate on the left) to the perception responses. Reaction times increased with perceptual instability. Further, a temporal preparation effect was apparent: Less temporal preparation is possible for the first stimulus within an ordered presentation sequence, resulting in longer reaction times.

**Fig 9:**
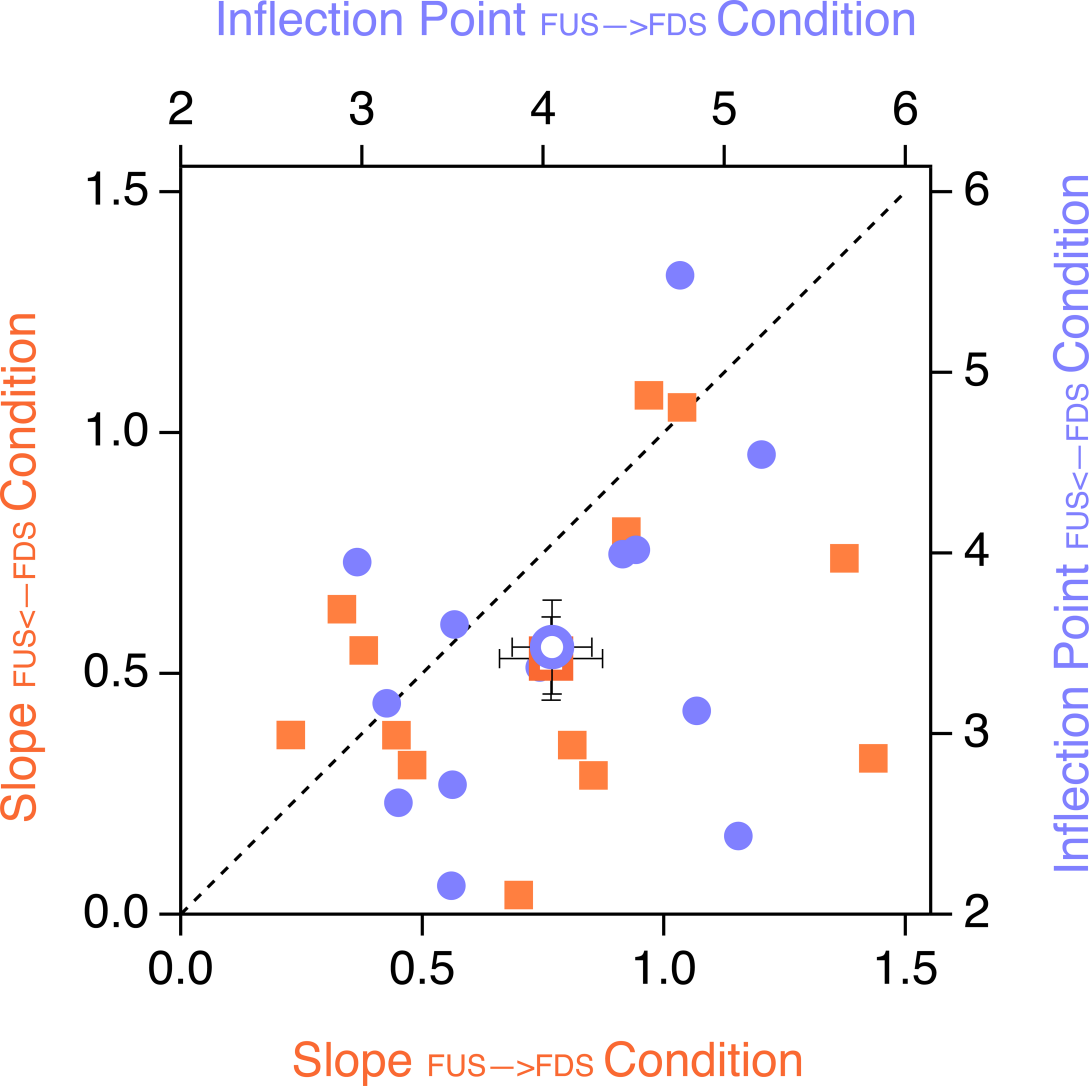
Scatter plot of the sigmoidal fit function parameters of the *FUS←FDS* and *FUS→FDS conditions* for the individual participants. Orange squares: Gradient values; cornflower circles: Inflection Point values. Open icons indicate grand means ± SEMs. Most cornflower icons are below the bisection line, indicating that the inflection points from the FUS←FDS condition show smaller values than from the *FUS→FDS condition*.

The perceived 3D orientations of the lattices changed as a sigmoidal function of the apparent back-layer’s luminance, as shown in the upper graph of Fig 8. The median R^2^s across participants and conditions is 0.97 with an interquartile range of 0.07, indicating very good fits of the sigmoid function also at the individual level. The repeated-measures ANOVA results for the variable *inflection point* (stimulus variant at 50% FU perception) revealed a main effect for the factor CONDITION (F_(2,11)_ = 6.768, p = 0.005). We observed no significant effect for the variable *gradient* although Fig 9 indicates a tendency for differences between presentation orders.

Scatter plots with individual inflection points and gradients of the sigmoidal fits are depicted in Fig 9. Ten of twelve subjects had larger inflection points for the *FUS→FDS* condition than the *FUS←FDS* condition, as indicated by the corresponding data located above the bisection line.

Post-hoc comparisons showed that, starting with stimulus S1 (*FUS→FDS condition)*, the inflection point occurred at a stimulus index number between S6 and S7, i.e. closer to S9 than when starting with S9 (the *FUS←FDS condition)* or in the *random condition* where the inflection point is located at S7. Bonferroni-Holm corrected post-hoc p-values are listed in Table 2.

**Table 2:** Corrected post-hoc results from Experiment 2.

comparison between conditions	p-value concerning the variable inflection point
*FUS←FDS vs*. *FUS→FDS*	0.03
*FUS→FDS* vs. random	0.01

#### Reaction times

In line with the percepts, reactions times differ between conditions, as indicated by the main effect for factor CONDITION (F_(2,24)_ = 5.580, p = 0.010). Further, the ANOVA indicated a cubic effect for the factor STIMULUS (F_(1,12)_ = 20.450, p = 0.001) as can be observed on the lower graph in Fig 8. Furthermore, we found a interaction effect between CONDITION and STIMULUS (F_(16,192)_ = 12.607, p < 0.001).

Inspection of Fig 8 reveals a similar pattern as already observed with the Mona Lisa stimuli in Experiment 1. Participants exhibited a temporal preparation effect [88,89] with longer reaction times for the first compared to subsequent stimuli in the two conditions with ordered stimulus presentations. We tested for temporal preparation effects and for different reaction times related to the perceptual bias (preferred FDP vs. non-preferred FUP) with a separate post-hoc ANOVA with the factors ORDER and BIAS. We found a main effect for the factor ORDER (F_(1,12)_ = 29.205, p < 0.001), showing that participants took longer to respond to the first presented stimulus than to the last (see Fig 8, bottom graph). No effect was observed for the factor BIAS.

#### Relations between Mona Lisa and lattice perception

For each of the two stimulus types we measured the reaction time costs of ambiguity by calculating the difference between the minimal and maximal reaction time across stimulus variants separately for the different experimental conditions. The results are listed in Table 3.

**Table 3:** Reaction time differences (maximum minus minimum across stimuli).

Condition	mean [ms]	standard error
*Mona Lisa*: *random*	259	20.2
*Mona Lisa*: *sad←happy*	244	20
*Mona Lisa*: *sad→happy*	303	30.4
*Necker lattice*: *random*	261	23.9
*Necker lattice*: *FUS→FDS*	276.1	27.5
*Necker lattice*: *FUS←FDS*	335.7	44.9

Comparing reaction time differences between Mona Lisa and the lattices (separate comparison between the random conditions and the averages of the two ordered conditions respectively) revealed no significant difference. This indicates similar reaction time costs of stimulus ambiguity for the two stimulus types.

We also found no significant differences between the sigmoidal gradients from the Mona Lisa sigmoid and those from the lattices (again separate comparisons between the random conditions and the averages of the two ordered conditions respectively). This indicates similar transition dynamics between the respective percepts with both stimulus types.

Finally we found neither a significant correlation (Person and Spearman) between Mona Lisa and lattices for the hysteresis distance nor for the average inflection points of the two different presentation orders.

## Discussion

In the present study we compared the perceptual processing of a Necker lattice as an example of low-level ambiguity, and the perceptual processing of the emotional face expression of Da Vinci’s Mona Lisa as an example of ambiguity at a higher processing level. We applied the hysteresis paradigm, presenting stimulus variants with different degrees of ambiguity in different sequences in order to disentangle the contributions of STM and LTM during the perceptual disambiguation.

For both stimulus types and all presentation sequences, perception described sigmoidal functions of the stimulus morphing parameters with comparable gradients across stimulus categories. Furthermore, we identified hysteresis effects for both stimulus types, reflecting STM contributions to the perceptual outcome: The morphing-parameter-value of that stimulus, evoking a perceptual transition from one to the other interpretations (i.e. location of the sigmoid inflection point) differed between the two ordered stimulus presentation conditions.

Interestingly, while the size of the hysteresis effect was similar between stimulus categories, its impact (sign) differed between them, showing priming patterns (positive hysteresis) for the lattice stimuli and adaptation patterns (negative hysteresis) for the face stimuli.

For the lattices, the random stimulus presentation condition revealed a strong LTM effect, as reflected in a perceptual bias favoring the front-side-down perspective (FDP). It is unclear whether LTM plays a role during face perception.

Finally, for both stimulus types, the reaction times for the perceptual decision largely correlated with the stimulus ambiguity, with similar reaction time costs of stimulus ambiguity of around 280 ms.

### Stimulus ambiguity at different processing levels

There is of course a significant difference between ambiguity in the Necker lattice and ambiguity in the emotional content of a face. Ambiguity in the case of the lattice can be described as essentially binary, because our perception typically oscillates between two clearly defined but mutually exclusive 3D interpretations, with high consistency of perceived angles across participants. Increasing the luminance of the apparent back layer of the disambiguated lattice variants (examples in Fig 1, third column) destabilizes perception, thus increasing the probability of a perceptual alternation, as reflected by the sigmoidal psychometric functions. However, the perceptual alternation always takes place between the two interpretations with clearly defined angle configurations of the lattice, without interpretations towards configurations with intermediate angular values.

This is different with the face stimuli: Although we are used to judging the emotional content of faces in our everyday life, the emotional state of a person being observed sometimes appears unclear and ambiguous. In fact, the different emotions and intensities that we can experience indicate a continuous rather than a binary scale. Further, emotion perception is a more complex task, involving social cognition and theory of mind (e.g., [32–34]). We have to refer to our own happiness and sadness experiences and relate them to certain facial features we identify in the person in front of us.

In the Mona Lisa variants, used in the present study, we solely manipulated the mouth curvature in order to manipulate the emotional face content. It is thus theoretically possible that our participants focused exclusively on the mouth curvature and ignored the rest of the face. As a consequence, social cognition and theory of mind may be not necessarily involved for the execution of the current task. Three arguments make this option less probable than a processing of the whole face:

- A perceptual decision based on the curvature of a line structure (Mona Lisa’s mouth) may be less effortful than a decision that needs a transition of a 2D retinal image into depth (lattices). Reaction times from the Mona Lisa experiment should thus be shorter than those from the lattice experiment. We found about equal reaction times (no significant difference in a post-hoc Wilcoxon test) in the case of Mona Lisa compared to the lattices (with a small tendency for longer reaction times in the case of Mona Lisa, as seen in Figs 6 and 8).
- Research on face perception indicates highly automatic (holistic) processing of faces (e.g., [91]). It may thus be more effortful to ignore (i.e. to purposely stop automatic face processing) than to process face information.
- In a current EEG study on the perception of faces with ambiguous and disambiguated emotional content, we again manipulated solely the mouth curvature of our face stimuli and participants decided between happy and sad face percepts. Our preliminary results show a clear face-specific N170 ERP component (a negative event-related potential, 170 ms and thus relatively early after onset of the face, and most prominent over the right fusiform face area). Importantly this face-specific N170 seems to be modulated by the perceived emotion. We regard this as a further–strong–indication that processing of faces and their emotion takes place even when the used stimuli would in principle also allow the ignorance of the whole face and the simple concentration on the line curvature of the mouth.

It is of course possible, that the adaptation effects we found with the Mona Lisa stimuli take place during processing of the mouth curvature. The perceived (adapted) mouth curvature may then be transmitted into face processing areas. In this case the source for perceptual ambiguity would lie at stages below face processing. One may thus ask whether it is appropriate to ascribe high-level ambiguity to the Mona Lisa task. Our arguments above speak against this objection, together with the comments of some of our participants who were convinced that not only mouth curvature but also the eyes and/or other face parts were manipulated between happy and sad face variants. Of course, such statements cannot be regarded as profound evidence.

We need further experiments to study whether the resolution of emotional ambiguities is just based on the analysis of individual face elements or whether something like emergent face features and/or emergent emotion features play a role.

Beyond the differences between Mona Lisa and lattice stimuli, we also found commonalities across the different levels of ambiguity. A most obvious one is that the relation between stimulus identity and percept is not one-to-one. Instead, one and the same sensory information can result in different interpretations (one-to-two or one-to-many).

In fact, theoretical approaches regard the perception of ambiguous figures as a low-level model of a cognitive decision process in higher-level planning and/or executive cognitive modules (e.g., [18,47–49]). Furthermore, our recent electrophysiological evidence indicates general properties of perceptual disambiguation beyond visual categories [52,53].

The hysteresis paradigm evokes perceptual alternations for both stimulus types externally by stepwise changes in a stimulus morphing parameter. It thus allows in principle direct comparisons between disambiguation of these very different types of sensory ambiguity. A meaningful quantitative comparison between stimulus categories, however, is not straightforward, in particular because we manipulated different types of morphing parameters for the two different stimulus categories. Comparisons thus require at least comparable scales of stimulus morphing parameters.

In pilot experiments we applied random stimulus presentation sequences and used the resulting sigmoids as reference functions for the choice of stimulus ranges. One most obvious criterion was equal numbers of stimulus variants. A second criterion was that for both categories the endpoint stimuli of the stimulus scale should evoke close to 100% perceptual probabilities. A third criterion was, that the departure from the 100% probability level should be evident at roughly the same stimulus numbers. All three criteria were fulfilled by our choice of the stimulus material, as can be seen in Figs 6 and 8. One similarity between stimulus types concerns the gradients of the resulting sigmoid functions.

### Other applications of the hysteresis paradigm

Another type of ambiguity with hysteresis effects has been observed in the behavioral dynamics of affordances, where perception and behavior are conceived as cyclically coupled (“action-perception cycle”). Affordances are potential activities of an agent whose different realizations are mutually exclusive. In this sense, the agent’s action, once it takes place, resolves the ambiguity of different possible reactions to the affordance.

Richardson et al. [75], Lopresti-Goodman et al. [76] and others studied the affordance “graspable” with respect to different behavioral dynamics, such as grasping an object unimanually or bimanually. In an experiment with a morphing parameter given by the ratio of object size vs hand span, distinct hysteresis effects as discussed so far have been found. Interestingly, there is a difference between positive and negative hysteresis, depending on whether the report on the kind of grasping was non-verbal (positive hysteresis) or verbal (negative hysteresis).

Lopresti-Goodman et al. [76] proposed a dynamical-system based model that accommodates this difference. Positive hysteresis can be successfully modelled by the behavior of an order parameter (unimanual vs bimanual grasping) as a function of a control parameter (ratio object size vs. hand span), By contrast, negative hysteresis requires an additional control parameter that is not externally manipulated but internally autoregulated. The action of this second control parameter is negative and repels the current order parameter into the alternative one. As this dynamics renders the current state less attractive with repetition, it resembles the feature that adaptation in bistable perception induces negative hysteresis. In this picture, lacking negative autoregulation (adaptation) favors positive hysteresis as the dominating behavioral pattern.

Exploiting the hysteresis paradigm to measure perceptual dynamics of ambiguous figures is not new. Research has been conducted in Necker cube ambiguity [23] and in motion perception ambiguity (Hock et al. [61,92,59], using von Schiller’s stroboscopic alternative motion (SAM or also called “motion quartet”, [7]): Two pairs of dots, located at the corners of an imaginary rectangle, are presented in alternation and induce the perception of either horizontal or vertical apparent motion (and in rare cases leftwards or rightwards rotation) as a function of the ratio between the vertical and the horizontal distance of the SAM dots (also called “aspect ratio”) [59,61,93–95]. At certain aspect ratios SAM motion perception becomes ambiguous with spontaneous alternations between the different motion direction interpretations. SAM motion ambiguity occurs not only in vision but also in touch [3,96–98].

In the study of Hock et al. [61], series of visual SAM variants were presented with either an ascending or descending aspect ratio in a hysteresis paradigm resembling the present one. Their results showed that the likelihood of perceptual alternation from the initially perceived motion direction to the alternative direction depended not only on the specific aspect ratio, but also on the presentation order and thus on the aspect ratios of the immediately preceding stimulus variants, similar to the hysteresis effects in our data. In particular, motion perception was primed by perceptual memory. Manipulating the luminance contrast between background and SAM dots, upon which the illusion of motion direction depends, is known to carry also a hysteresis effect in the perception of motion [92].

Pisarchik et al. [99] recently presented the Necker cube in a hysteresis paradigm with a continuously changing stimulus morphing parameter (contrast of cube edges). Interestingly they found both positive and negative perceptual hysteresis as a function of externally introduced noise (varying background luminance) and of the change-velocity of the morphing parameter. Negative hysteresis resulted mainly as a function of increasing noise, which was confirmed by a non-linear deterministic energy model, applied to the empirical data.

### The role of short-term memory during perceptual disambiguation

Our perceptual system uses information stored in our memories in order to interpret the a priori incomplete, noisy and to varying degrees ambiguous sensory information and to construct stable and reliable percepts. Any present percept can thus be influenced to a certain amount by what we have seen immediately before. As a consequence, the identity of the stimulus variant perceived as most ambiguous (at the sigmoid’s inflection point) changed depending on perceptual history and therewith on the stimulus presentation order. The perceptual hysteresis effect quantifies this influence of the immediately preceding stimulus and can thus be interpreted as the contribution of STM to the perceptual process.

Assuming comparable ranges of stimulus variants, the contribution of STM to the disambiguation of the lattices is about half of the contribution for the disambiguation of Mona Lisa’s emotion (factor-2-relation). The more ambiguous the sensory information is, the larger becomes the influence of STM contributions. This influence reached up to 34% in the case of Mona Lisa and up to 17% in the case of the lattices, as indicated by the peaks of the green difference traces in Figs 6 and 8. Comparing this perceptual variance depending on STM between stimuli reveals again a factor-2-relation, which we will turn to below.

Besides the observed relation between STM influences, we found qualitatively opposite STM impacts on the two stimulus categories. In the case of the ordered presentations of the lattice stimuli, the preceding stimulus variants exhibit a *priming* impact on perception, increasing both the total time and the stimulus range over which the initial perceptual interpretation remains unchanged. This results in a shift of the sigmoid function in direction of the stimulus presentation order (Fig 8, upper graphs, which is in line with the prediction from Fig 2B).

In case of the Mona Lisa variants’ ordered presentations, the preceding stimuli show an *adaptation* impact on perception of the current stimulus, decreasing both the total observation time and the range over which the initial perceptual interpretation stays unchanged. This results in a shift of the sigmoid function in a direction opposite to the stimulus presentation order (Fig 6, upper graphs, in line with the prediction from Fig 2A).

Priming and adaptation effects have already been reported for the Necker cube and other ambiguous figures (e.g., [24–31]). They have been explained as functions of the stimulus pre-exposure’s durations: Following the brief pre-exposure of an unambiguous version of the stimulus (up to 20 seconds), the observer is more likely to report the same configuration (priming) when perceiving the ambiguous stimulus. The opposite effect (adaptation) would follow after longer presentations (e.g., [24,26]).

In line with those studies, a priming effect has been observed after a 1200 ms exposure to previous lattice variants. Interesting in this context are findings from van Rooij et al. [23]. They also used lattice stimuli and a hysteresis paradigm similar to the one in the present study. However, they reported hysteresis effects with an adaptation impact, i.e. opposite to our priming findings. This apparent contradiction may be explained in the following way: In the lattice experiment reported here we presented nine stimuli, four disambiguated variants favoring FDP and four favoring FUP. Our average “pre-exposure time” (the presentation time of disambiguated stimulus variants until the perceptual alternation happens) thus lasts about 4.8 seconds (i.e. 4 x 1.2 s). Van Rooij et al. presented 17 lattice stimuli, i.e. eight variants disambiguated in either direction. As a consequence, their participants were exposed twice as long to disambiguated lattice variants, which may have turned the priming impact into an adaptation impact.

But how can we integrate the Mona Lisa findings in this context? The perception of faces is known to be special. During our lives we look much more often into a human face than at any other object. Faces are known to be processed faster and they evoke stronger physiological responses (e.g., [100,101]).

Priming and adaptation are also well-known phenomena in the face perception literature [28,37,37–46]. It is interesting in the context of the present results that face adaptation starts to occur already after very brief stimulus pre-exposures of 160 ms [43,45]. Furthermore, such adaptation effects can last for up to 24-hours [45], especially with more familiar faces, such as Mona Lisa’s [39,40]. In our study, each stimulus was presented for 1200 ms, which is more than enough time to adapt the neural network underlying face perception.

But there is also another factor that can heavily influence the outcome of ambiguous figure perception: Several studies presented ambiguous figures discontinuously with short gaps between stimulus presentations and found strong influence of the gap duration on the perceptual dynamics of ambiguous figures with both adaptation and priming effects (e.g. [102–108]). Overall, it is obvious that stimulus-on- and -off-times are important factors that may influence the quality of perceptual outcomes also in the present paradigm. The different stimulus types used in the current experiments may show different time constants for one or even both of those factors. Future experiments need to systematically test their influence. Further, future modeling work may help to better understand how on- and off-times, stimulus-specific perceptual time constants and neural background activity (noise) are related to nonlinear system behaviour (e.g., [99]).

### The role of long-term memory during perceptual disambiguation

Looking at the Necker cube, observers are typically biased towards the front-side-down perspective (FDP) (e.g., [20–23]). Such a priori perceptual biases can be interpreted as the influence of lifetime perceptual statistics from everyday experience stored in LTM: We look down at objects much more often than looking up to objects. If the sensory evidence is maximally ambiguous this kind of LTM may become a decisive factor during perceptual disambiguation.

The hysteresis paradigm allows quantification of this LTM impact: Presenting the stimulus variants in random sequence, STM’s contribution can be averaged out across repetitions. As a consequence the physically ambiguous Necker lattice should also be the perceptually most ambiguous stimulus with parity of the two perceptual interpretations. Accordingly, the inflection point of the resulting sigmoidal function should be located exactly at this physically most ambiguous reference stimulus (see Fig 2 and the related explanations). We interpret the horizontal shift of the sigmoid curve related to the random condition away from this expected location as the contribution of the a priori bias due to LTM. The perceptually most ambiguous stimulus turned out to be located between two lattice variants, already containing strong cues favouring the FUP interpretation (Fig 1, right column stimulus S3). These cues appear to be necessary to compensate for the strong a priori perceptual bias due to LTM. Moreover, the probability of FDP for the physically ambiguous Necker lattice (Fig 1, right column, stimulus S5) is at around 80% instead of the expected 50%. The effect of LTM can thus be quantified by a shift of about 30%.

Another interesting finding is that the sigmoid function of the *FDS*→*FUS* condition is superimposed on that from the random condition. Given that the STM contribution is averaged out in the random condition and assuming that the LTM influence is also present with the ordered stimulus presentation (see Eqs 1–3 and the related explanations in the introduction) this superposition of sigmoid functions can be interpreted in the following way: In the case of the lattice stimuli, our results from the random condition indicate a considerable LTM influence. The intensity of the 3D cues (gray scale of the apparent lattice back-layer) that compensates for this strong LTM influence (to reach parity between the two interpretations) attained such strength that no additional STM influence from the *FDS*→*FUS* ordered stimulus presentation condition was efficacious. As a result, the sigmoid function from the corresponding ordered condition was not shifted further, but instead remained nearly coincident with the sigmoid function from the random condition. Consequently, the hysteresis distance appears not due a double-sided STM influence, as with the Mona Lisa stimuli (see Eq 3 in the introduction), but due to a one-sided STM influence. This explains the factor-2-relation between Mona Lisa results and lattice results.

In summary, the influence of LTM during perceptual disambiguation of the lattice stimuli is reflected in two aspects. First, the sigmoid inflection point from the random stimulus presentation condition is not located at the physically most ambiguous Necker lattice. Second, memory influence during perceptual disambiguation is limited. If a certain threshold is reached, the sigmoids from the two ordered stimulus presentation conditions are no longer symmetrically positioned equidistant to the sigmoid from the random condition (compare Fig 2A and 2B). In the case of the lattice stimuli we found the extreme case of an almost coincidence of the corresponding sigmoids.

The participants’ preference for happy face percepts, as reported in the literature (e.g., [9,84–86]), predicts the presence of an a priori bias for the perception of the Mona Lisa variants as well. However, the situation is different here, compared to the lattice stimuli, because we do not have a “physically” most ambiguous reference stimulus, like the Necker lattice in Experiment 2. Moreover, we have recently observed that the perceptual processing of a facial emotion like Mona Lisa’s strongly depends on and quickly adjusts to the total range of emotional stimulus variants [9].

For the Mona Lisa stimuli, the sigmoid functions from the ordered stimulus presentation sequences are located at about equal distance to the sigmoid from the random presentation condition. Because of the absence of a clearly defined reference stimulus we conclude that a hypothetical threshold for memory influence during perception, as in the case of the lattice stimuli, has not yet been reached with the Mona Lisa stimuli. We thus cannot determine whether LTM influences the perception of Mona Lisa’s emotional face expression.

### Visual ambiguity, temporal preparation and reaction times

The reaction times are in a range expected from previous studies (e.g. [109,52]). A common pattern found in both stimulus categories is the longer reaction times for the first stimuli in the ordered stimulus presentation conditions compared to the last, even though the perceptions for both the first and the last stimuli reach 100% probabilities, thus stable percepts. We explain this pattern with the well-known ‘temporal preparation effect’ or its absence for the initial stimuli in a sequence. The temporal preparation effect is observed as a time benefit when the stimulus occurrence can be temporally anticipated [87–89].

In our experiment, the observer’s response to the first stimulus variant could not profit from the temporal regularity underlying the exposure of the other stimuli in the hysteresis paradigm. Its temporal anticipation was thus more difficult, which most probably explains the absence of a temporal preparation effect and thus the longer reaction times. In the random stimulus presentation condition, each of the nine stimuli (per stimulus category) was about equally often the first stimulus (across repetitions). As a result, the averaged reaction times (across repetitions) for each of the presented stimuli contains trials with and without temporal preparation effects, resulting in slightly longer overall reaction times, compared to the ordered conditions, together with slightly larger variances. Both can be seen in Figs 6 and 8 (lower graphs).

In addition and occasionally superimposed we find a second reaction time effect: Reaction times for the perceptual decision largely correlate with the degree of ambiguity of the stimulus. We conjecture that, if the level of ambiguity of the sensory information is high, the observer tends to rely more on the contribution from STM and LTM, and the perceptual process may be more effortful and thus it takes longer to respond than to the unambiguous stimuli. In fact, the reaction times for perceptual judgments increased by about 280 ms from maximally disambiguated stimulus variants to the perceptually most ambiguous stimulus variants. We found very similar values for the two different stimulus categories, indicating equal “temporal costs” or a similar number of processing steps to resolve these different types of stimulus ambiguity.

### Da Vinci’s original Mona Lisa is always happy

Mona Lisa’s emotional face expression is considered worldwide as an emblematic example of emotional ambiguity [35]. A possible explanation for Mona Lisa’s ambiguity may be the observers’ spatial resolution of the image. Livingstone [110] was the first to propose an inverse relation between perceived happiness of Mona Lisa and the spatial resolution of the image’s mouth area (she discussed this in terms of spatial frequencies). Amongst others [111,112], the study of Soranzo & Newberry [113] provided empirical evidence supporting Livingstone’s proposal: Their participants perceived Mona Lisa as being less content when looking at the painting from a close distance, and more content from a greater distance. The size of the Mona Lisa face region in Soranzo & Newberry’s far observer distance condition was about 1.35° x 1.5° visual angle. In this condition the observers reported maximum values of contentment (75% of the cases) in Mona Lisa’s facial expression.

In our recent study [9], as well as in the present study, the size of Mona Lisa’s face region was about 5 times larger (6.8° x 7.8°) and should thus have been perceived as less happy compared to Soranzo & Newberry’s study [113] (assuming a similarity of happiness and contentment). However, in our study participants gave close to 100% happiness responses. A similar divergence from the above prediction can be observed in Kontsevich & Tyler’s study [112].

Of course, the cited studies differ not only in the perceived spatial resolution of the Mona Lisa images but also in several other aspects, like the range of stimulus variants, the way the stimuli had been manipulated (e.g. gray scale vs. color images etc.). Some or all of these factors could have had a potential influence on participants’ perception. Overall, the divergence in results may also be evidence that Mona Lisa’s mystery has indeed not yet been completely resolved, even though she can appear unambiguously happy in a certain experimental setting.

## Summary and outlook

Several theoretical approaches assume principal generalities in the processing of ambiguous information, independent of whether ambiguity occurs at a lower visual level or at higher levels involving social cognition, theory of mind or verbal communication. In the present study we investigated ambiguity between different processing levels, employing the ambiguous Necker cube and the enigmatic Mona Lisa as examples. We investigated the interplay between processing of sensory information and memory contributions during the perceptual inference process and found both commonalities and differences: (1) For both stimulus categories perception follows highly similar sigmoidal functions of the stimulus morphing parameters with similar gradients. (2) The amount of STM contribution is reflected in positive and negative hysteresis effects that can be quantitatively determined by the sigmoids’ inflection points. (3) The temporal cost of resolving stimulus ambiguity (reaction times) are in a similar range for both stimulus categories, even though the impact of STM is qualitatively opposite.

Perceptual hysteresis does not only allow a comparison between apparently incomparable processing levels of visual ambiguity. It also reduces the well-known inter- and intra-individual variability typically found with reversal rates and stability durations in classical ambiguous figures (e.g., [114,115]).

In the case of the lattice stimuli, low-level ambiguity resolution occurs during the 3D interpretations of the individual lattice edges. Although there is an high-level ambiguity in Mona Lisa’s emotional expression, the critical parameter in the present experiment is the mouth curvature, a low-level feature. This may be a possible explanation for some of the commonalities in the results for the two stimulus types.

Psychiatric patients with autism spectrum disorder or schizophrenia often exhibit perceptual abnormalities, like a local bias in sensory processing (e.g., [116]) or hallucinations and delusions (e.g., [117]). Current explanations assume a maladaptive integration of sensory processing with prior knowledge from perceptual memory during the perception in these patients (e.g., [118,119]). The hysteresis paradigm allows systematic quantitative testing of this hypothesis, as was also proposed by Martin et al [120]. The possibility to compare between lower and higher levels of stimulus processing is particularly important in this context, because ambiguity resolution in social cognition contexts seems to be more affected in such patients than at lower visual levels. Related studies are currently being conducted.

## Supporting information

S1 Data Data file containing percept responses and averaged reaction times (+ standard deviations) of the individual participants and experimental conditions. (XLSX)
